# Stress management strategies for NP practice

**DOI:** 10.1097/01.NPR.0000000000000144

**Published:** 2024-01-25

**Authors:** Denise R. Felsenstein

**Affiliations:** **Denise R. Felsenstein** is a continuing education manager at Wolters Kluwer Health, an adjunct lecturer for the Health and Exercise Physiology Department at Ursinus College, and is the owner/founder of Teach the Nurse, LLC.

**Keywords:** breathing techniques, distress, guided imagery, humor, laughter, meditation, mindfulness, stress, stress management, stress reduction strategies, stressors

## Abstract

Knowledge of stress management strategies is helpful to NPs in clinical practice, as they frequently encounter patients or patients' family members who require assistance in managing acute stress. Patients or patients' family members may experience a high level of stress due to health factors such as pain, life-changing diagnoses, treatment options viewed as undesirable, and/or poor medical prognoses. In addition, healthcare visits, hospitalization, diagnostic tests, surgical procedures, and other treatments can cause stress for some patients. NPs should therefore be well informed about stress management strategies to be able to effectively educate and provide compassionate care for their patients. This article reviews four strategies designed to assist patients and/or their family members in reducing stress to allow for a more positive experience during a healthcare visit or hospitalization.

## Abstract 2

Knowledge of stress management strategies is helpful to NPs in clinical practice, as they frequently encounter patients or patients' family members who require assistance in managing acute stress. Patients or patients' family members may experience a high level of stress due to health factors such as pain, life-changing diagnoses, treatment options viewed as undesirable, and/or poor medical prognoses. In addition, healthcare visits, hospitalization, diagnostic tests, surgical procedures, and other treatments can cause stress for some patients. This article reviews four strategies designed to assist patients and/or their family members in reducing stress to allow for a more positive experience during a healthcare visit or hospitalization.

**Figure:**
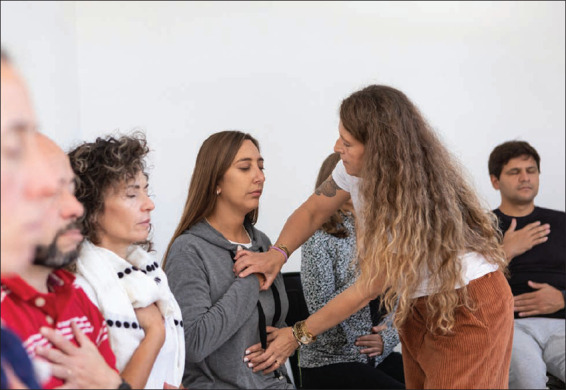
No caption available.

The definition of stress is often personal, with its perception and experience depending on each individual's unique perspective, among many other variables. When a stimulus is viewed as a threat, an automatic response known as stress occurs.^1^ The American Psychological Association (APA) Dictionary of Psychology defines stress as a physical or mental reaction that occurs due to internal or external stressors.^2^ A stressor is defined as a stimulus that causes stress.^3^ A stressor is the catalyst that is perceived as a threat and that in turn causes a cascade of events, known as the stress response, to occur. Seaward explains that the stress response becomes activated by all types of threats, not just physical intimidations.^4^ These perceived threats, which vary individually, trigger the stress response, commonly known as “fight or flight.”

There are a variety of types of stress. Eustress is considered a “good” stress that helps the individual achieve something that they desire. An example of eustress is working hard to achieve an athletic goal. Distress (“stress”) occurs due to a situation that the individual perceives negatively. An example is a patient's receipt of a diagnosis that can have life-changing ramifications. The duration of stress can be short (“acute stress”) or long (“chronic stress”).^5^ Acute stress results from a negative but short-lived situation that induces stress. Chronic stress occurs when the stress response is triggered for a considerable amount of time due to a situation that does not quickly resolve. The amount of time is not defined and can range from weeks or months to years, depending on the situation. The primary caregiver of a spouse with Parkinson disease, for example, may experience chronic stress.

## Perception

According to survey data collected in 2022, the stress level of Americans is reported to be 20% higher than the average in other countries.^6^ In the same survey, 55% of American participants reported that they were feeling stressed during the day, and 57% felt that their stress caused them to feel paralyzed. Finally, 49% of respondents indicated that their coping strategy entailed enduring the stress they were experiencing.

The Yerkes-Dodson law, developed in 1908 by Harvard physicians Robert M. Yerkes and John Dillingham Dodson, identified the relationship between performance level and amount of stress.^5^ A particular situation does not cause stress; rather, how an individual interprets a situation induces the response. For example, if two people experience the same event at the same time, one may perceive this event as a threat that initiates a stress response, whereas the other person may not view the event as a threat and therefore may not experience stress.

## Pathophysiology and symptoms

Olpin and Hesson explain that a person's ability to manage stress can either lead to a positive or negative outcome.^5^ The amount and type of stress endured by an individual can either be motivating, if the demand is a reasonable one for the body to handle, or it can be overwhelming, as in response to a demand that cannot be met. The body's capacity to manage stress in a healthy way greatly impacts the emotional and physical response to a stressor. A high frequency and significant amount of stress on an individual lead to depletion of the body's resources, reducing the ability to respond or function in an effective manner.

The stress response is thought to be triggered when the body's senses provide information to the amygdala, which identifies a given situation as a threat. The hypothalamo-pituitary-adrenocortical axis response is then initiated through the release of corticotropin-releasing hormone from the hypothalamus, leading the anterior pituitary gland to release adrenocorticotropic hormone, which activates the adrenal cortex to release the glucocorticoid cortisol.^7^ Cortisol initiates gluconeogenesis, providing energy for the stress response.^7^ The hypothalamus sends activation signals to the sympathetic nervous system (SNS), and the adrenal medulla releases epinephrine and norepinephrine. This process initiates symptoms such as tachycardia, tachypnea, elevated BP, muscle tension, and diaphoresis.^5^ Increased inflammation can also occur due to immune suppression, production of fewer lymphocytes, and release of proinflammatory cytokines.^8^ This activation of the SNS also inhibits digestion. If the stress response trigger is effectively resolved, the release of epinephrine and norepinephrine from the adrenal medulla is inhibited, as is release of cortisol from the adrenal gland cortex.

## Rationale for using stress management strategies

The rationale for using stress management techniques is that they have the potential to stop the stress response from causing fight-or-flight symptoms and to allow the body to return to a state of homeostasis. Stress reduction strategies activate the parasympathetic nervous system (PNS), thereby facilitating the return to homeostasis by reducing BP, pulse, and respiratory rates; initiating muscle relaxation; and resuming digestive and immune functions.^5^

Stress can be exacerbated in healthcare situations. Zisopoulou and Varvogli found that use of stress reduction techniques helps to reduce both stress levels and disease-related symptoms.^1^ Stress management strategies are easy to teach and learn, with patients of varying ages and abilities capable of adopting them, and they have clear benefits.

## Teaching stress management strategies

Patients may be amenable to learning positive coping strategies to mitigate any acute stress that they may feel while attending a healthcare visit or experiencing hospitalization. NPs should be prepared to assist and teach patients stress reduction strategies when symptoms of distress are evident. Teaching a simple breathing technique, guided imagery activity, meditation/mindfulness exercise, or laughter/humor strategy can be time efficient and relatively easy, as no tools are needed. A benefit is that patients can achieve effective symptom reduction in a short time frame.

When handling a situation in which a patient's stress response has been triggered, use the following steps. First, assess the situation and determine what has occurred to initiate the patient's stress response. Then, develop a plan to reduce the stress-related symptoms or to address the underlying cause of the stress. Next, implement the planned stress management strategy to decrease the patient's response to the stressor. Last, evaluate whether the technique used was effective for reducing the patient's stress. If the strategy did not successfully reduce the patient's stress, determine whether the technique could be combined with another strategy to increase its effectiveness or if using a different stress reduction strategy may be needed.

## Impact of stress management strategies

In a systematic review and meta-analysis of stress management intervention effectiveness for college students, Amanvermez and colleagues found that, when compared with control conditions, stress management programs were effective; this is similar to findings of other meta-analyses performed on this topic with noncollege student populations.^9^ After analyzing the literature, Amanvermez and colleagues determined that medium- or longer-term interventions for stress management might have more benefit than shorter-term interventions. This recent meta-analysis found that the study interventions had a great effect on cortisol levels of students. A randomized study by Hoogland and colleagues described and evaluated the efficacy of implementing the Spanish-Language Self-Administered Stress Management Training (SL-SAT) intervention, which includes three stress management techniques (deep breathing, progressive muscle relaxation and guided imagery, and coping self-statements), in Spanish-speaking Hispanic and Latina women starting chemotherapy for cancer diagnoses.^10^ The researchers compared the SL-SAT participants with usual care participants, who only received an educational booklet about coping with chemotherapy. This study revealed that both groups experienced improvements in emotional well-being, suggesting that Latina populations receiving chemotherapy might benefit from attention from a Spanish-speaking interventionist in a group-based format over a longer period of time.

## Stress management strategies

In this section, four stress management strategies (breathing techniques; guided imagery; meditation and mindfulness; and laughter and humor) are outlined, with an overview of how each strategy works. A summary of benefits and examples of application are also provided. This section is intended to guide the NP through implementing these strategies in practice. Table 1 provides a brief overview of the information found in this section.

**Table 1.** Overview of stress management strategies^4^,^5^,^11^-^14^,^16^

Stress management strategy	Description and explanation of strategy	Guidance or instructions for NPs	Strategy benefits
Breathing techniques	The patient breathes slowly and deeply to focus their thoughts solely on inhaling, taking a pause, and exhaling. By breathing in a very focused and controlled manner, the patient can distract their mind from negative thoughts, reduce their respiratory and pulse rates, and increase their oxygenation.	*Diaphragmatic breathing:* Ask the patient to inhale slowly through their nose, allowing air to fill the upper then lower lobes of their lungs by contracting the diaphragm downward and causing the abdomen to expand and rise. Then, have the patient hold their breath for a few seconds. Finally, ask the patient to control their exhalation of air slowly through their mouth, allowing the diaphragm to relax upwards and the abdomen to return to its resting state. *Four-square (or box) breathing:* Advise the patient to inhale slowly through the nose to the count of 4 seconds, hold their breath to the count of 4 seconds, slowly exhale through their mouth to the count of 4 seconds, and rest for a count of 4 seconds. Repeat the cycle.	Reduces pulse rate Lowers BP Reduces muscle tension Reduces cortisol levels
Guided imagery	The patient experiences an imaginary environment that they feel is a relaxing setting as well as the accompanying sensations of the imagined environment. Guided imagery is performed by using soothing words, providing detailed explanations of restful settings, and discussing sensations they would experience to allow for full engagement, helping to achieve relaxation of the patient's mind and body.	Ask the patient to close their eyes and focus on taking slow, deep breaths. Then, in a soft voice, describe a step-by-step journey into an imaginary relaxing environment, such as walking on a beach, strolling around a flower garden, or fishing at a lake. Engage all the patient's senses in the imaginary scene to promote a feeling of relaxation during a healthcare visit, test, or procedure. Spend the amount of time that is needed or is possible to guide the patient through the imagined setting.	Reduces symptoms related to the activation of the SNS Activates the PNS
Mindfulness and meditation	The patient experiences detached awareness while observing their surroundings. They focus on being objective about their experience and fully present while performing an easy everyday activity, such as walking, eating, or drinking. This distracts the patient's mind from stressful thoughts as they fully engage in the present moment by experiencing the sensations of the activity.	Encourage the patient to be fully present by experiencing and focusing on all their senses during a daily activity, such as slowly walking, eating, or drinking. Advise the patient to experience this activity in a focused but objective manner with no associated judgment.	Reduces pain Promotes a feeling of peacefulness Fosters feelings of transcendence Reduces SNS arousal Enhances PNS engagement
Laughter and humor	When laughing or engaged in humor, the patient experiences a eustress stimulus, which produces a positive emotion. Laughter creates a physical response that exercises the facial and abdominal muscles, which causes the patient to take deeper breaths and reduces stress-related hormone levels.	There are a variety of humor types and numerous ways to engage patients in laughter. Some examples include keeping a notebook with a variety of funny cartoons (intended to suit different humor types) to share with patients; keeping some short funny videos on a laptop or cell phone to share with patients; keeping a journal with a variety of jokes; and engaging patients with one's natural wit. For all suggestions, be sure to use humor that is not offensive to others.	Decreases feelings of anger and fear Exercises the muscles, then helps enhance relaxation of muscles after laughter is over Increases tolerance to pain Reduces levels of stress hormones Stimulates blood circulation Elevates mood Increases pleasure in life Improves relationships Provides feelings of connection with others Reduces negative thoughts

Abbreviations: PNS, parasympathetic nervous system; SNS, sympathetic nervous system.

### Breathing techniques

NPs can suggest use of breathing techniques in many situations, given that these exercises are simple to use and effective in quickly reducing stress-related symptoms. Diaphragmatic breathing, for example, helps patients to concentrate on their breath instead of on stressors, leading to stress symptom reduction. To perform this strategy, the NP should instruct the patient to place one hand on the chest and one on the abdomen. Then, the patient should be advised to focus on keeping the upper hand still by avoiding movement of the chest and raising the lower hand with each inhalation by expanding the abdomen as the diaphragm contracts. Instruct the patient to hold their breath for a few seconds and then to exhale slowly through their mouth, allowing the abdomen to return to a relaxed position. Repeat this cycle as needed to assist the patient in reducing feelings of stress. In terms of efficacy, a quantitative systematic review by Hopper and colleagues studying the effectiveness of diaphragmatic breathing for reducing adult physiologic and psychological stress found that physiologic biomarkers of stress decreased and psychological stress was reported to decrease based on self-report tool data.^11^

Another breathing technique that may help patients to reduce their stress levels is four-square (or box) breathing. To perform this strategy, advise the patient to inhale slowly through the nose to the count of 4 seconds, hold their breath to the count of 4 seconds, and then slowly exhale through their mouth to the count of 4 seconds. Finally, rest for a count of 4 seconds before repeating the cycle.

The case scenario at the end of this article explains how an NP might use diaphragmatic breathing and four-square (or box) breathing to reduce a patient's stress. To enhance the experience and engage the senses, the NP could consider adding some relaxing instrumental music to the room or could use aromatherapy by applying one drop of lavender essential oil on a cotton ball and placing it in the vicinity. If still ineffective, another technique, such as guided imagery, could be employed next.

### Guided imagery

The use of guided imagery helps patients to focus on imagined sensations to achieve a state of relaxation. Guided imagery exercises entail creation of a scenario that brings the patient's mind into an imagined environment that they find relaxing while encouraging them to focus on their perception of each of their senses in that environment. For example, the NP might have the patient focus on imagining a beach setting, flower garden, or lake environment as part of the guided imagery technique. The patient can close their eyes while the NP describes taking a step-by-step journey into a calm and peaceful setting. Ask the patient to imagine what it smells like in this imagined environment. Ask what they hear and feel. Ask if they have a taste in their mouth in the imagined setting. The NP can include all senses in the journey to create a deeper sense of relaxation as the patient becomes more engaged in the guided imagery scenario. A guided imagery session can last however long is possible or until the NP sees a reduction in the patient's stress. For example, consider using this technique when a patient's BP reading is high. Initiate a brief guided imagery session for 5 minutes before measuring BP again to determine if the patient has white coat syndrome or needs continued BP workup for possible hypertension. Guided imagery was shown in a 2016 study of its use for stress reduction among pregnant adolescents to provide benefits: Using the Perceived Stress Measure-9 tool to measure levels of stress, Flynn and colleagues found that the study population experienced short- and long-term stress reduction through this intervention.^12^

### Meditation and mindfulness

The NP can use meditation to assist patients with relaxation for procedures, medication administration (such as chemotherapy), or other healthcare-related situations that can induce a patient's stress response. To implement a simple mantra meditation technique, the NP first should have the patient focus on their breath using a diaphragmatic breathing technique. If the patient's mind begins to wander, advise them to bring their mind back to their breath as a grounding base for meditation. The patient can either close their eyes or keep them semiopen, gazing down. Next, assist the patient in choosing a mantra, such as “I am relaxed.” Ask the patient to repeat the mantra in a slow and focused way, either in their head or in a soft whisper. Mantra meditation can be used for a time frame that works best for the given situation to help to reduce SNS-related stress symptoms.

Mindfulness meditation is another technique that can be used to reduce patient stress. Mindfulness meditation involves instructing the individual to act as an objective observer who engages in normal daily activities in a nonjudgmental manner, fully aware of all experiences and sensations. To assist a patient in practicing mindfulness, the NP can guide the patient in being present and fully aware of their environment by accepting what they are experiencing from an objective observer's viewpoint without attaching any judgment to the situation.

Many studies address the use of mindfulness for reducing stress in patients with a variety of diagnoses. In a study conducted in patients with cancer experiencing stress in an outpatient setting, a mindfulness meditation-based stress reduction program showed that the mindfulness meditation intervention effectively reduced symptoms of stress among patients of different ages and sexes with a wide variety of cancer diagnoses and stages.^13^ In a 2023 study conducted by Ashraf and colleagues, a mindfulness-based stress reduction therapy (MBSRT) program was implemented to help patients diagnosed with irritable bowel syndrome (IBS).^14^ In this study, MBSRT entailed patients focusing on noticing details around them without reacting to triggers of stress, instead observing the situation in an objective manner without passing judgment.^14^ Findings of this study showed that participants receiving the MBSRT intervention had a higher quality of life and reduced IBS symptoms as compared with the control group.^14^

A separate study of a mindfulness-based stress reduction (MBSR) strategy required participants to examine and experience the many details of three raisins by using their senses of smell, taste, and touch, then by slowly eating them one at a time while being fully immersed in the experience.^15^ The NP can use a similar strategy in a healthcare setting using other types of food, such as pretzels, or having the patient drink a cup of water while fully focusing all their senses on slowly eating or drinking. This practice can help the patient detach from negative thoughts by distracting the mind. While eating, the patient should focus on the sensations associated with slowly chewing the pretzel; with softening it and reducing it in size; and finally with swallowing it. When drinking water, the patient can focus on how it feels to hold the cup in their hands, to have the water touch their lips and teeth, to have the water slowly enter their mouth, to swish the water in their mouth, and then to swallow it, noticing how it feels as it slides down the esophagus into the stomach.

### Laughter and humor

The use of humor and laughter is a stress reduction strategy that can assist the NP in creating a relaxing atmosphere in the healthcare setting. When a patient finds something humorous and laughs, it reduces the level of their stress hormones (cortisol and adrenaline) and increases the release of brain endorphins (including dopamine) to gain a positive emotional mood and physical relaxation.^16^ These changes occur whether the patient forces the laughter or genuinely laughs. Seaward described using different types of humor such as parody, satire, and slapstick for stress reduction.^4^ Providing appropriate humor during a patient visit can quickly reduce a stressful situation, assist with relaxation, and initiate a trusting relationship. The NP can describe funny situations or have cartoons available that are humorous to reduce a patient's care-related stress. Choose humor that others will not find to be offensive, hurtful, or inappropriate. Avoid using sarcasm, since this type of humor can be hurtful.^4^ Use of laughter and humor quickly reduces stress and builds trusting relationships with patients, as it can increase connectedness between the patient and provider and establish a higher level of patient comfort with the provider and healthcare setting. Benefits of laughter include reducing stress hormones, increasing pain threshold and tolerance, reducing anxiety and depression, enhancing self-esteem, and improving interpersonal interactions and feelings of closeness.^5^ When an individual laughs, an arousal phase occurs that stimulates the body by initially increasing BP, pulse, and respiratory rates. A resolution phase follows this arousal phase of laughter, thereby returning the body to a resting state and resulting in feelings of relaxation. Tension is released from muscles during the laughter process, and endorphins in the brain are elevated. This creates a eustress response. Use of humor and laughter can be a helpful coping mechanism for patients receiving palliative care for terminal illnesses and their caregivers, helping to increase quality of life.^16^

## Case scenario

Maria is a 40-year-old woman who arrives at the clinic for an annual gynecologic visit. She is tearful and having difficulty verbalizing her distress. She is triaged by the nurse and placed into an exam room for the NP to provide care. When the NP enters the room and starts reviewing Maria's medical history form with her, Maria begins sobbing uncontrollably. The NP asks her to explain why she is upset, but Maria is unable to stop crying long enough to speak. The NP realizes that using a stress management strategy for Maria could be helpful. The NP teaches Maria a diaphragmatic breathing technique by first demonstrating it and then performing it simultaneously with Maria. Next, the NP asks Maria to watch her perform four-square breathing before guiding Maria through the process. Maria engages in four-square breathing until she starts relaxing and feeling more comfortable. After six cycles of four-square breathing, Maria is able to explain the reason for crying. She states she had a routine Papanicolaou (Pap) test last year in a private physician's office. Maria further explains that the physician called her on the phone 2 weeks later to inform her that the human papillomavirus (HPV), which is associated with cervical cancer, had been found in her specimen and that she needed to return for another Pap test the following year. Maria states that she is afraid of having a Pap test today because she feels that the result will show that she is dying of cervical cancer. The NP provides Maria with patient education about HPV, cervical cancer risk, and Pap testing. The NP advises Maria to have the Pap test performed now, explaining that results are delivered within a 2-week period. In the meantime, the NP recommends that Maria continue to use the diaphragmatic or four-square breathing technique whenever she begins feeling overwhelmed with stress. Maria states her understanding of the information provided and agrees to have the Pap test performed with HPV testing. Maria leaves the clinic feeling less stressed than when she entered. Ten days later, the NP receives Maria's testing results. The Pap test is normal with no HPV present. The NP calls Maria to advise her of these results. Maria is thrilled with this news and states her appreciation of the NP's care.

## Conclusion

Learning stress management strategies can assist NPs in providing compassionate care for patients during times of stress at healthcare facilities. Patients experience different levels of stress during such situations as healthcare visits, hospitalizations, diagnostic tests, and treatment procedures. Each situation may be perceived as stressful based on a patient's prior negative experience or possible anticipation of pain or suffering. Within minutes, the NP can reduce a patient's stress by teaching them techniques to use to stop SNS-related symptoms, trigger the PNS, and stop the negative feedback loop that causes the stress response to occur.

Review the four stress management strategies discussed in this article and keep them in an imaginary toolbox for use in a healthcare setting to provide exceptional patient care. Continue to learn new strategies to add to this imaginary toolbox. Assess the situation before planning and implementing a stress reduction strategy that would work well for the scenario to help the patient to cope with health-related stress.
